# How to Go to Bed

**Published:** 1869-08

**Authors:** 


### HOW TO GO TO BED

In freezing Winter-time. Do it in a hurry, if there is no fire in the room; and there ought not to be, unless you are quite an invalid.

But if a person is not in good health, it is best to undress by a good fire; warm and dry the feet well; draw on the stockings again; run into a room without fire; jump into bed, cuddle up, with head and ears under cover for a minute or more, until you feel a little warmth; then uncover your head; next, draw off your stockings, straighten out, turn over on your right side, and go to sleep.

If a sense of chilliness comes over you on getting into bed, it always will do an injury; and its repetition increases the ill effects, without having any tendency to "harden" you. Nature abhors violence. We are never shocked into health. Hard usage makes no garment last longer.

